# Myocardial Infarction in Murine Models of Obesity and Related Metabolic Disorders: The Role of Inflammation

**DOI:** 10.3390/ijms262311663

**Published:** 2025-12-02

**Authors:** Lotte Geerkens, Stefan Janssens, Senne De Groote, Matic Pusovnik, Wouter Oosterlinck, Uwe Himmelreich

**Affiliations:** 1 Biomedical MRI, Department of Imaging and Pathology, KU Leuven, Herestraat 49, 3000 Leuven, Belgium; lotte.geerkens@kuleuven.be (L.G.); matic.pusovnik@kuleuven.be (M.P.); uwe.himmelreich@kuleuven.be (U.H.); 2 Department of Cardiovascular Sciences, KU Leuven, Herestraat 49, 3000 Leuven, Belgium; stefan.janssens@uzleuven.be (S.J.); senne.degroote@kuleuven.be (S.D.G.)

**Keywords:** murine models, myocardial infarction, ischemia–reperfusion, obesity, inflammation

## Abstract

Cardiovascular disease (CVD) remains a leading cause of mortality worldwide, with myocardial infarction (MI) being a major contributor, particularly among individuals with obesity, a prevalent risk factor. Inflammation plays a key role in both MI and obesity, as well as in ischemia–reperfusion injury (I/R), the paradoxical cardiac injury response triggered by reperfusion. The complex cellular and molecular interplay between these risk factors in the context of MI remains incompletely understood. Preclinical research using murine models is crucial for studying disease mechanisms, identifying therapeutic targets, and advancing drug development. Despite promising preclinical findings, clinical translation of therapies targeting inflammation has been largely disappointing. A major shortcoming is the predominant use of healthy mice without comorbidities in studies of inflammation in MI. A deeper understanding of inflammatory signaling in mouse models of obesity and related metabolic disorders may help bridge the gap between preclinical research and successful clinical application. In this review, we focus on the specific role of inflammation in MI murine models with obesity and related metabolic disorders. We aim to provide a better understanding of the apparent variability in their cardiac injury phenotype, address the existing controversies in reported data, and highlight directions for future research.

## 1. Introduction

Cardiovascular disease remains the leading cause of death in Europe and worldwide, with myocardial infarction (MI) accounting for the majority of deaths [1,2]. MI is caused by the sudden occlusion of the coronary artery, restricting blood flow to the myocardium, leading to ischemic injury and contractile dysfunction in the affected myocardial territory [1,3]. In the event of an acute MI, prompt reperfusion, typically via percutaneous coronary intervention (PCI) or coronary artery bypass grafting, is of crucial importance to reduce infarct size and improve clinical outcome [4,5]. At the cellular level, early initiation of inflammation mediated by infiltrating white blood cells plays a crucial role in the clearance of necrotic cardiac cells and in tissue repair. However, restoration of blood flow to the ischemic region also results in paradoxical myocardial damage in a process termed ischemia–reperfusion (I/R) injury. I/R injury is characterized by the activation of distinct pro-inflammatory and pro-oxidant pathways, and contributes to impaired wound healing, infarct extension, and adverse cardiac remodeling [3,6,7,8,9,10].

**Figure 1:**
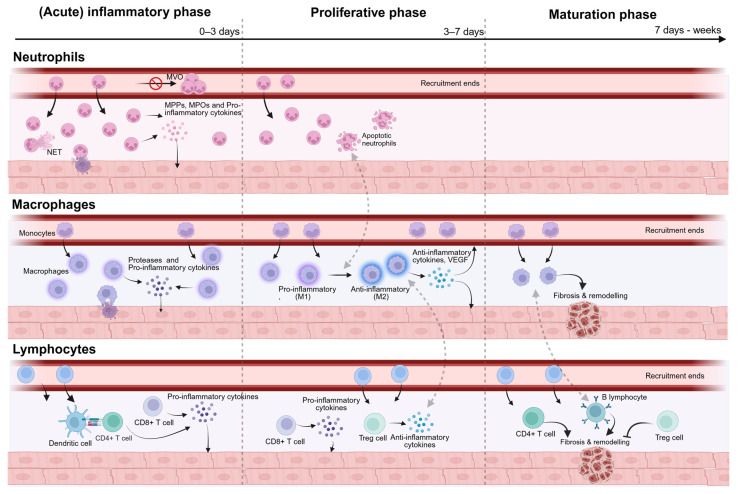
**Schematic overview of inflammation following myocardial infarction.** Illustrating the dynamics of neutrophils, macrophages, and lymphocytes across three phases: the (acute) inflammatory phase, the proliferative phase, and the maturation phase. Dashed arrows indicate interactions between the different cell types. Abbreviations: MVO Microvascular Obstruction, MMPs Matrix metalloproteinases, MPOs Myeloperoxidases, NET neutrophil extracellular trap, VEGF Vascular endothelial growth factor, Treg cell Regulatory T cell. Created in BioRender. Geerkens, L. (2025) https://BioRender.com/zclkfcx (accessed on 26 August 2025).

Different immune cells are activated following MI, mediating the inflammatory response (Figure 1) [11].

**Figure 2:**
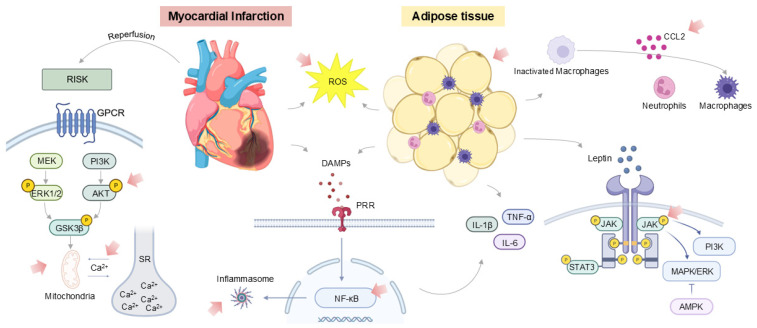
**Inflammatory pathways in myocardial infarction affected by obesity and related disorders.** In acute MI, damage-associated molecular patterns (DAMPs) are released from injured myocardium and bind to and activate pattern recognition receptors (PRRs) on resident cardiac cells. PRR-signaling leads to NF-kB and inflammasome activation with subsequent generation of pro-inflammatory cytokines, including IL-6, TNF-α, and IL-1β. At the onset of reperfusion, the Reperfusion Injury Salvage Kinase (RISK) pathway is activated, which consists of a cluster of prosurvival kinases, including PI3K-Akt, and MEK-ERK1/2, activated upon restoration of blood flow after ischemia to protect cardiomyocytes from apoptotic cell death. Obesity leads to a chronic low-grade inflammatory state with elevated cytokine levels in adipose tissue and systemically (leptin, IL-6, IL-1β, TNF-α), which activate circulating immune cells and cardiac resident cells. In the context of MI, increased leptin expression and release activate the leptin receptor, which lacks intrinsic kinase activity and recruits JAK(2) to its intracellular domain, which in turn can activate several intracellular canonical signaling pathways, depending on the context. In the early phase of I/R, leptin exerts cardioprotection via PI3K and STAT3 activation. In later phases of MI, chronic leptin signaling shifts toward pro-inflammatory and pro-fibrotic pathways via STAT3–JAK2 and via MAPK/ERK. In contrast, AMPK activation in obesity reduces MAPK activation during I/R and improves cardiomyocyte mitochondrial function. The pink arrows indicate where the negative obesity-related impact occurs. Abbreviations: CCL2 C-C Motif Chemokine Ligand 2, ROS reactive oxygen species, IL-1β Interleukin-1 beta, TNF-α Tumor necrosis factor alpha, IL-6 Interleukin-6, JAK/STAT Janus kinase/signal transducer and activator of transcription signaling pathway, MAPK Mitogen-activated protein kinase, PI3K Phosphoinositide 3-kinase, AMPK adenosine monophosphate-activated protein kinase, P phosphorylation, DAMPS Damage-associated molecular patterns, NF-κB Nuclear factor kappa B, RISK reperfusion injury salvage kinase, GPCR G protein-coupled receptor, MEK MAPK/ERK kinase mitogen-activated protein kinase/extracellular signal-regulated kinases, ERK1/2 extracellular signal-regulated kinase, AKT Protein kinase B, GSK3β Glycogen synthase kinase-3 beta, SR sarcoplasmic reticulum, PRR Pattern Recognition Receptor. Created in BioRender. Geerkens, L. (2025) https://BioRender.com/q4bugfd (accessed on 26 August 2025).

These immune cells, on one hand, may contribute to cardiac repair but could also exacerbate reperfusion injury by inducing an excessive inflammatory response [12,13]. Inflammation also plays a crucial role in obesity and related disorders, which are significant risk factors for MI [14]. Generally, outcomes after MI are less favorable in the setting of obesity. Obesity often coexists with conditions like diabetes, hypertension, and dyslipidemia, all of which predispose to post-MI complications [15,16,17]. However, the relationship between obesity and MI outcome is complex. Obesity is associated with a state of low-grade chronic inflammation and increases the risk of developing CVD, including MI [14,18]. This inflammatory state is primarily driven by the recruitment and activation of pro-inflammatory macrophages [14]. Besides macrophages, other types of immune cells, such as neutrophils and lymphocytes, contribute to these inflammatory processes [11]. Chronic inflammation in obesity and related metabolic disorders plays a role in insulin signal transduction and may lead to insulin resistance and β-cell dysfunction, eventually predisposing to the development of long-term complications, including CVD [14,19]. While inflammation triggers metabolic dysregulation, adipose tissue in obesity generates pro-inflammatory cytokines, leading to cardiomyocyte dysfunction. Epicardial adipose tissue releases adipokines, which have detrimental effects on cardiac metabolism, promote cardiomyocyte inflammation, redox imbalance, and myocardial fibrosis [15,20,21]. Obesity-related factors that contribute to this enhanced cardiac fibrosis include increased ROS production, M1 macrophage polarization, increased leptin synthesis leading to activation of MAPK and JAK/STAT signaling pathways, and insulin resistance triggering PI3K/Akt signaling [22]. Immune cells play an essential role in modulating inflammation during and after MI [23], especially in the context of obesity and concomitant chronic inflammation (Figure 2). Hence, immune cells are promising targets for therapeutic interventions to limit infarct size and I/R injury, prevent heart failure, and reduce mortality.

Some studies have indicated that obesity is associated with better prognosis following MI, a concept known as the obesity paradox [15,24,25]. The term “obesity paradox” refers to the observation that obesity in patients with CVD is associated with lower mortality compared to normal-weight patients. Although this paradox has been repeatedly reported in various CVD and therapeutic interventions, including PCI, it remains enigmatic and controversial [26,27]. Indeed, in incident disease cohorts or better-designed studies with robust and comprehensive adjustments to reduce bias, the paradox weakens or disappears [28,29], raising major concerns about the neglect of statistical confounders [26,27]. Inflammation has been proposed as one of the contributing factors, although its precise role and underlying mechanisms remain unclear [30].

Preclinical research using animal models of MI is essential for understanding underlying cellular mechanisms and molecular pathways with therapeutic potential, for identifying and evaluating innovative therapeutic interventions, and for advancing drug development. Experimental mouse models are widely used in cardiovascular research because of well-established hemodynamic, imaging, and pathological parameters and the availability of various genetically modified mouse strains, which allow for detailed investigation into disease-specific pathophysiological mechanisms [31,32]. Despite the promising results reported in multiple preclinical studies testing pharmacological and mechanical therapies, including strategies that target inflammation, clinical translation in patient trials of acute MI has been generally disappointing [5,6,33,34]. Therefore, it is important to improve our understanding of the inflammatory signaling in mouse models used to study MI in the context of obesity and related metabolic disorders, since inflammation is a key player in MI and obesity [3,34]. This might improve the bench-to-bedside transition of preclinically tested interventions to improve clinical outcomes after MI.

## 2. Mouse Models of Myocardial Infarction

Preclinical animal models play a crucial role in advancing our understanding of MI and developing therapeutic strategies. Before mice emerged as the animal of choice to study MI [32,35], most MI experiments were performed on large animal models [35,36,37,38,39]. New surgical, hemodynamic monitoring, and imaging techniques have made cardiac interventions on small animals feasible. Additionally, rodents, in particular mice, are cost-effective, easy to handle, have a high reproduction rate, and offer many genetically modified strains providing numerous benefits over large animals [32,40,41].

**Figure 3:**
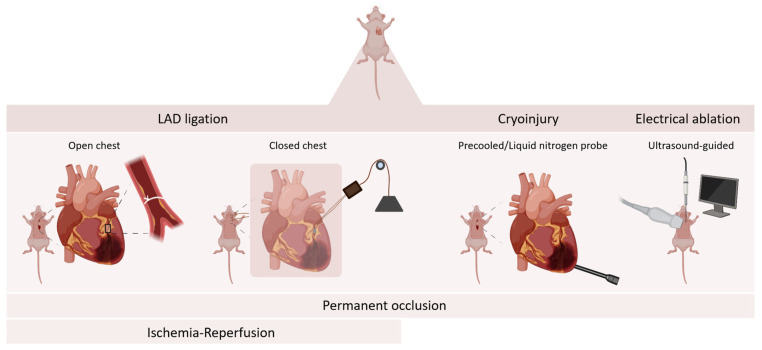
**Overview of myocardial infarction mouse models.** Surgical ligation of the left anterior descending artery (LAD) is performed through the placement of a suture around the LAD. LAD ligation can be performed in an open chest or closed chest model. LAD ligation allows both permanent occlusion and ischemia–reperfusion. Ablation through cryoinjury is induced by applying a precooled or liquid nitrogen probe to the epicardial surface at a specific location for a precise time period. Electrical ablation is a minimally invasive approach using ultrasound-guided high-frequency electricity to coagulate the LAD. Cryoinjury and electrical ablation only allow permanent occlusion. Created in BioRender. Geerkens, L. (2025) https://BioRender.com/tta7dff (accessed on 26 August 2025).

Different methods are used to induce MI in mice (Figure 3). The most commonly used method involves either permanent or transient surgical ligation of the left anterior descending artery (LAD), replicating the pathophysiologic mechanisms observed in myocardial ischemia and resulting in myocardial infarction [35]. Permanent ligation causes irreversible ischemia and transmural necrosis of the LAD-dependent myocardial territory, i.e., the anterior wall and apex of the LV. This model represents the patient population in whom prompt reperfusion could not be established or in whom reperfusion failed. The extensive myocardial necrosis following permanent ligation enables investigation of the scarred region with infarct wall thinning and subsequent left ventricle (LV) dilation, which in turn contributes to heart failure progression [32,35,42]. In contrast, transient coronary ligation models of variable duration more closely mimic the clinical setting of PCI in which the patients’ blood flow is timely restored, resulting in a reduced infarct size and increased survival [43,44]. Consequently, the infarct size after prompt I/R is smaller compared to permanent occlusion because a part of the area at risk (AAR) is salvaged by reperfusion. Coronary occlusion duration of more than 1–2 h causes irreversible damage and shows comparable results as permanent ligation. Compared to the permanent occlusion model, I/R results in more limited cardiac remodeling with less pronounced infarct wall thinning and loss of LV shape and function.

In contrast, sudden restoration of coronary blood flow following transient ligation introduces the phenomenon of I/R injury, causing further damage to the myocardium, as also observed in the clinical setting [32,35,45]. This paradoxical injury is governed by altered inflammatory responses compared to the permanent ligation model. Cardiac cell death following MI releases damage-associated molecular patterns (DAMPs), triggering the innate immune system to produce pro-inflammatory cytokines and chemokines, recruiting different subpopulations of infiltrating leukocytes, generating a strong inflammatory reaction [46]. This inflammatory cellular response is carefully orchestrated depending on the injury model. Certain cell types exhibit a temporally shifted immune response, such as the macrophage population, which peaks 4 days earlier in the reperfusion model compared to the permanent ligation [47]. For both permanent occlusion and I/R models, evaluation during the first week after ischemia is commonly used to assess inflammatory responses, with days 1, 3, 5, and 7 being the most frequently reported time points. For long-term follow-up, a 4-week endpoint is most typical, although assessments at 2, 8, and 12 weeks are reported as well [45]. Furthermore, the I/R models enable ischemic conditioning of the heart, a cardioprotective mechanism introduced to reduce I/R injury. Ischemic conditioning involves brief repetitive cycles of coronary or vascular occlusion–reperfusion before, during, or after reperfusion, referred to as pre-, per-, and postconditioning [48,49]. To study inflammatory changes upon MI, a closed-chest approach might be beneficial as it eliminates the confounding immune response induced by the thoracotomy [50,51].

Other, less commonly used murine MI models include ablation methods through cryoinjury or electrical damage. Cryoinfarction is induced by applying a precooled or liquid nitrogen probe to the epicardial surface at a specific location for a defined period of time. This approach enhances reproducibility, given the ability to control the size, shape, and location of the infarct. However, in contrast to the hypoxia-induced apoptosis caused by LAD occlusion, cryoablation leads to cardiomyocyte cell membrane disruption and necrosis. More recently, a minimal-invasive approach of electrical ablation using ultrasound-guided high-frequency electricity to coagulate the LAD has been described [52]. Given the distinctive way of cell death upon ablation, these models do not reflect the typical infarct-induced inflammation [32,45].

## 3. Myocardial Infarction in Murine Models of Obesity and Related Metabolic Disorders

**Figure 4:**
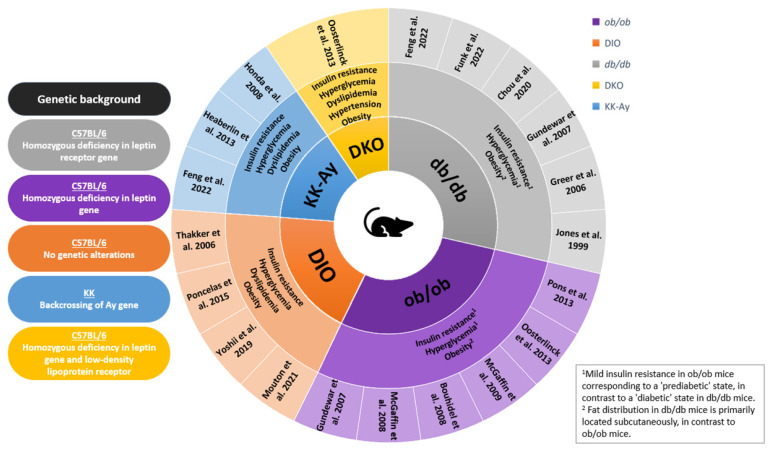
**Overview of the different murine models of obesity and related disorders in MI studies.** The primary ring represents the genetic background, with the specific genetic alterations depicted on the left for every murine model. The secondary ring holds information on the characteristic phenotypic traits for every strain. In the box, the relative differences in properties between the *ob*/*ob* and *db*/*db* mouse models are highlighted. The tertiary ring shows in which publications the corresponding mouse model was used in the context of I/R surgery. Additionally, the proportions of the segments illustrate how many articles there are for each mouse model, with bigger segments reflecting a greater number of articles. Abbreviations: *db*/*db* mice homozygous for the diabetes spontaneous mutation (Lepr^db^), DIO Diet Induced Obesity, DKO Double *ob*/*ob* and low-density lipoprotein receptor^−/−^ knockout, KK-Ay mice heterozygous for the yellow spontaneous mutation (Ay) on a KK strain background, *ob*/*ob* mice homozygous for the obese spontaneous mutation, Lep^ob^. Feng et al. [59], Funk et al. [60], Chou et al. [61], Gundewar et al. [62], Greer et al. [63], Jones et al. [56], Pons et al. [64], Oosterlinck et al. [58], McGaffin et al. [65], Bouhidel et al. [53], McGaffin et al. [66], Mouton et al. [67], Yoshii et al. [68], Poncelas et al. [69], Thakker et al. [55], Heaberlin et al. [57], Honda et al. [70].

The primary challenge in experimental MI studies is to select the most representative preclinical model that reflects the most important comorbidities that drive the clinical phenotype of MI. However, the majority of preclinical studies have focused on young, healthy animals without comorbidities and thus are not representative of the patient population at risk for MI. Hence, there is an unmet need for experimental animal models that consider associated risk factors, including older age, female gender, obesity, diabetes, hyperlipidemia, and hypertension, which represent the heterogeneity observed in patients with MI. Each of these risk factors can individually affect infarct size and the outcome of pharmacological or mechanical interventions [32,53,54]. Few studies have investigated the inflammatory response and subsequent cardiac remodeling after I/R injury in mouse models of obesity, diabetes, and related disorders. Table 1 provides an overview of these studies, including details on ischemia and reperfusion duration, as well as information on infarct size, cardiac function, survival, and the inflammatory or immune markers that have been assessed. The two most frequently used genetically modified mouse models for MI studies in obesity and obesity-related disorders are leptin-deficient *ob*/*ob* mice, showing characteristics of obesity and insulin resistance, and diabetic *db*/*db* mice with leptin receptor-deficiency [55,56]. Other related models include diet-induced obese (DIO) mice [55], diabetic and obese KKAy mice, a cross between diabetic KK and lethal yellow (Ay) mice [57], and mouse models of the metabolic syndrome (Double *ob*/*ob* and low-density lipoprotein receptor^−/−^ knockout: DKO) with features of diabetes, obesity, dyslipidemia, and atherosclerosis [58] (Figure 4).

**Table 1:** Overview of MI studies in mouse models of obesity, diabetes, and related disorders. Abbreviations: MI Myocardial Infarction, PL Permanent Ligation, I/R Ischemia–Reperfusion, I Ischemia, WT Wild-Type, PI Post Infarction, IPostC Ischemic Postconditioning, AN/AAR Area of Necrosis/Area At Risk, AAR Area At Risk, DIO Diet-Induced Obesity, TMS treatment N,N,N-Trimethylsphingosine chloride, HTN Hypertension, DKO Double *ob*/*ob* and low-density lipoprotein receptor^−/−^ Knockout, SGLT1 Sodium/glucose cotransporter 1, PMN polymorphonuclear neutrophils, LV Left Ventricle, RB40.34 mAb Monoclonal Antibody directed against P-selectin, GAME46 mAb Monoclonal Antibody directed against CD18 Ca(i) Intracellular Calcium, ECM Extracellular Matrix, MG53 Mitsugumin 53, hSA Human Serum Albumin, rhMG53-WT Recombinant Human Wild-Type MG53 protein, rhMG53-C14A E3 Recombinant Human E3 ligase-dead mutant MG53 protein.

Paper	Mouse Model	Outcome	Sex	Total Number	Age	PL or I/R	Groups & Size	Weight (g)	Infarct Size-Timepoint	Cardiac Function	Survival	Inflammatory/Immune Markers
McGaffin et al., 2008 * [66]	*ob*/*ob*	MI increases **leptin receptor activation** & leptin repletion in *ob*/*ob* improves outcome	male	n = 110	5–6 weeks	PL	WT ad libitum (n = 16) WT ad libitum sham (n = 10) Ob ad libitum (n = 18) Ob ad libitum sham (n = 11) Ob food restrict (n = 15) Ob food restrict (n = 9) Ob leptin replete (n = 13)	28.1 ± 0.5 51.8 ± 0.5 27.5 ± 0.5 27.5 ± 0.5	6.16 ± 0.07 mm^2^ 6.19 ± 0.10 mm^2^ 6.17 ± 0.15 mm^2^ 6.25 ± 0.18 mm^2^ (short axis, 4 weeks PI)	↓ ↓↓ ↓↓ ↓ (4 weeks PI)	75% 44% 46% 69%	STAT3 ↑, timp1, hsp70 ↑ STAT3 =, timp1 =, hsp70 = STAT3 =, timp1 =, hsp70 = STAT3 ↑, timp1 ↑, hsp70 ↑
McGaffin et al., 2009 * [65]	*ob*/*ob*	**Leptin signaling** after post-MI activates anti-apoptotic genes through **STAT-3,** reducing caspase-3 activity, limiting cardiac apoptosis	male	n = 16	5–6 weeks	PL	WT Ob ad libitum Ob food restrict Ob leptin replete	28.1 ± 0.5 51.8 ± 0.5 27.5 ± 0.5 27.5 ± 0.6	6.16 ± 0.07 mm^2^ 6.19 ± 0.10 mm^2^ 6.17 ± 0.15 mm^2^ 6.25 ± 0.18 mm^2^ (short axis, 4 weeks PI)	↓ ↓↓ ↓↓ ↓ (4 weeks PI)	75% 44% 46% 69%	Apoptosis ↑, CD45+ cells ↑ Apoptosis ↑↑↑, CD45+ cells ↑↑↑ Apoptosis ↑↑↑, CD45+ cells ↑↑↑ Apoptosis ↑, CD45+ cells ↑
Oosterlinck et al., 2013 [58]	*ob*/*ob* DKO (MS)	Protection by **IPostC** against I/R injury remains but might be reduced in *ob*/*ob* mice and DKO mice	male & female	n = 80	24 weeks	I/R 30 min I	WT (n = 12) WT + IPostC (n = 6) WT sham (n = 6) *ob*/*ob* (n = 10) *ob*/*ob* + IPostC (n = 10) *ob*/*ob* sham (n = 8) DKO (n = 6) DKO + IPostC (n = 6) DKO sham (n = 6)	27 ± 1 63 ± 1 60 ± 2	52% 31% 58% 44% 54% 41% (AN/AAR, 60 min PI)	↓↓ * ↓ * ↓↓ ↓ ↓↓ * ↓ * (60 min PI) +1&10 weeks *	100% 100% / / 17% 67%	/
Bouhidel et al., 2008 [53]	*ob*/*ob*	**IPostC** protection against I/R injury is lost in *ob*/*ob* mice, likely due to reduced RISK pathway activation	male	n = 33	8–10 weeks	I/R 30 min I	WT (n = 9) WT + IPostC (n = 9) *ob*/*ob* (n = 8) *ob*/*ob* + IPostC (n = 7)	25.5 ± 0.7 23.4 ± 0.3 48.2 ± 1.2 46.0 ± 1.9	44 ± 3% 40 ± 3% 41 ± 4% 27 ± 2% (%AAR, 24 h PI)	/	/	P-Akt, P-ERK1/2, P-p70S6K1, P-AMPK ↑ P-Akt, P-ERK1/2, P-p70S6K1, P-AMPK ↑↑↑ P-Akt, P-ERK1/2, P-p70S6K1, P-AMPK ↑↑ P-Akt, P- ERK1/2, P-p70S6K1, P-AMPK ↑↑
Pons et al., 2013 [64]	*ob*/*ob*	**Treadmill exercise** induces cardioprotection against MI and restores pro-survival signaling pathways	male	n = 33	5–10 weeks	I/R 30 min I	WT (n = 7) WT + exercise (n = 9) *ob*/*ob* (n = 8) *ob*/*ob* + excercise (n = 9)	25.0 ± 0.6 24.2 ± 1.0 42.8 ± 0.6 44.8 ± 0.7	43 ± 3% 17 ± 2% 58 ± 3% 19 ± 1% (%AAR, 24 h PI)	/	/	P-Akt, P- ERK1/2, P-p70S6K1, P-AMPK, P-GSK3β, Ca^2+^ ↑ P-Akt, P- ERK1/2, P-p70S6K1, P-AMPK, P-GSK3β, Ca^2+^ ↑↑ P-Akt, P- ERK1/2, P-p70S6K1, P-AMPK, P-GSK3β, Ca^2+^ ↑ P-Akt, P- ERK1/2, P-p70S6K1, P-AMPK, P-GSK3β, Ca^2+^ ↑↑
Gundewar et al., 2007 [62]	*ob*/*ob* *db*/*db*	Cytoprotective effect of **TMS treatment** upon I/R is lost in *ob*/*ob* and *db*/*db*	date not reported	n = 106	8–10 weeks	I/R 30 min I	WT (n = 13) WT + TMS (n = 30) *ob*/*ob* (n = 12) *ob*/*ob* + TMS (n = 23) *db*/*db* (n = 11) *db*/*db* + TMS (n = 17)	26 ± 1 48 ± 1 44 ± 1	50.83 ± 1.89% 17.32 ±2.11% 53.41 ± 4.41% 34.24 ± 3.54%, ~59% (from graph) ~53% (from graph) (%AAR, 24 h PI)	↓↓ ↓ / / / / (72 h PI)	/	PKC-δ translocation to mitochondria ↑↑ PKC-δ translocation to mitochondria ↑ / / PKC-δ translocation to mitochondria ↑↑ PKC-δ translocation to mitochondria ↑↑
Thakker et al., 2006 [55]	DIO	DIO mice showed increased **inflammation** and impaired **healing**, associated with adverse remodeling	male & female	n = 32	30–32 weeks	I/R 1 h I	WT male (n = 8) WT female (n = 8) DIO male (n = 8) DIO female (n = 8)	27.45 ± 1.26 23.54 ± 0.57 46.41 ± 2.42 36.82 ± 0.87	(data on collagen deposition and amount of fibrosis provided) (7 days PI)	↓ (7 days PI)	79.3% 67.8%	MIP-1α, MIP-1β, MIP2, MCP-1, IP-10 ↑ IL-6, IL-10, Osteopontin, TGF-β1, TGF-β3 ↑ MIP-1α, MIP-1β, MIP2, MCP-1, IP-10 ↑↑ IL-6, IL-10, Osteopontin, TGF-β1, TGF-β3 ↑↑
Mouton et al., 2021 [67]	DIO	Obesity lowers survival but improves cardiac function and **metabolism** in surviving **normotensive** mice	male & female	n = 90	12–24 weeks	PL	WT male (n = 9) WT female (n = 8) DIO male (n = 16) DIO female (n = 13) DIO male + HTN (n = 24) DIO female + HTN (n = 20)	/	~51% ~40% ~42% ~41% ~49% ~39% (from graph, 7 days PI)	↓↓ ↓↓ ↓ ↓ ↓↓↓ ↓↓↓ (7 days PI)	89% 75% 56% 54% 29% 35%	P-Akt, P-ACC, P-PDH, P-AMPK, PPAR-gamma, PGC-1α = P-Akt, P-ACC, P-PDH, P-AMPK, PPAR-gamma, PGC-1α = P-Akt ↑↑, P-ACC ↑↑, P-PDH =, P-AMPK ↑, PPAR-gamma ↑, PGC-1α = P-Akt ↑, P-ACC ↑, P-PDH =, P-AMPK =, PPAR-gamma =, PGC-1α = P-Akt =, P-ACC ↑, P-PDH =, P-AMPK =, PPAR-gamma =, PGC-1α = P-Akt =, P-ACC =, P-PDH =, P-AMPK =, PPAR-gamma =, PGC-1α =
Poncelas et al., 2015 [69]	DIO	DIO attenuates postinfarct myocardial **remodeling** and dysfunction in adult B6D2F1 mice	male	n = 52	26–30 weeks	I/R 45 min I	WT (n = 16) WT sham (n = 10) DIO (n = 16) DIO sham (n = 10)	37.8 ± 1.7 48.3 ± 0.1	34.06 ± 9.35% 15.57 ± 4.63% (area of fibrosis)	↓↓ ↓ (7 and 28 days PI)	92.4% (general survival)	P-Akt, P-GSK3β ↑ P-Akt, P-GSK3β ↑↑
Yoshii et al., 2019 [68]	DIO	**SGLT1** contributes to cardioprotection during the acute phase of I/R injury via enhanced **glucose transport**	male	n = 28	8–20 weeks	I/R 30 min I	WT (n = 6) WT + plorizin (SGLTi) (n = 6) DIO (n = 8) DIO + plorizin (SGLTi) (n = 8)	~31 ~46 (from graph)	32.3% ± 2.2% 52.5% ± 3.5% 60.2% ± 1.5% 71.8% ± 4.0% (MI area/ventricular area, 40 min PI)	↓ ↓↓ ↓↓ ↓↓↓ (40 min PI)	/	GLUT-4 ↑↑, SGLT1 ↑ GLUT-4 ↑, SGLT1 ↑
Greer et al., 2006 [63]	*db*/*db*	**Varying durations** of myocardial **I/R** in *db*/*db* mice influence survival and cardiac function (heart failure)	male	n = 134	10 weeks	I/R 30, 45 or 60 min I	WT − 30’ I (n = 12) WT − 45’ I (n = 16) *db*/*db* − 30’ I (n = 55) *db*/*db* − 45’ I (n = 34) *db*/*db* − 60’ I (n = 17)	24 ± 0.4 49 ± 0.4	\	= = = ↓↓ / (28 days PI)	100% 88% 71% 53% 18%	/
Jones et al., 1999 [56]	*db*/*db*	Reperfusion injury is worse in *db*/*db* mice, likely due to **PMN-driven** inflammation, as **CD18** neutralization reduces infarct size, but independent of **P-selectin**	male	n = 42	date not reported	I/R 30 min I	WT (n = 15) *db*/*db* (n = 14) *db*/*db* + RB40.34 (n = 6) *db*/*db* + GAME46 (n = 7)	/	27.2 ± 3.1 % 56.3 ± 2.8 % 47.2 ± 9.4 % 34.4 ± 8.1% (%AAR, 2 h PI)	/	88% 58%	Myocardial neutrophil (PMN) ↑ Myocardial neutrophil (PMN) ↑↑↑ Myocardial neutrophil (PMN) ↑↑ Myocardial neutrophil (PMN) ↑
Chou et al., 2020 [61]	*db*/*db*	**Ranolazine** improves Ca(i) dynamics and conduction inhomogeneity in I/R injury	female	n = 29	23–30 weeks	I/R 15 min I	*db*/*db* (n = 7) *db*/*db* + Ranolazine (n = 7) db/+ (n = 8) db/+ + Ranolazine (n = 7)	55.0 ± 7.8 59.6 ± 12.0 30.1 ± 3.9 31.9 ± 4.2	/	/	/	/
Funk et al., 2022 [60]	*db*/*db*	**Sarcomere function** in the remote zone is impaired after I/R due to failed compensatory mechanisms and worsened **calcium handling**	male	n = 32	10–12 weeks	I/R 60 min I	db/+ (n = 10) db/+ + I/R (n = 6) *db*/*db* (n = 10) *db*/*db* + I/R (n = 6)	27.5 ± 1.1 46.0 ± 2.0	37 ± 2% 36 ± 2 % (Ischemic area LV free wall) (24 h PI)	= ↓ = ↓↓ (24 h PI)		P-ERK1/2 =, P-PKCα =, P-PLN(m)(T17) =, Col3a1 = P- ERK1/2 =, P-PKCα ↑, P-PLN(m)(T17) ↑↑, Col3a1 = P- ERK1/2 =, P-PKCα ↑, P-PLN(m)(T17) =, Col3a1 ↑↑↑ P- ERK1/2 ↑, P-PKCα =, P-PLN(m)(T17) ↑, Col3a1 ↑↑↑
Heaberlin et al., 2013 [57]	*Kkay*	Kkay mice have a reduced post-MI survival but **improved cardiac function** through reduced inflammation, ECM accumulation, and neovascularization in the infarct region	male & female	n = 49	24–32 weeks	PL	WT (n = 7) WT + MI (n = 10) *Kkay* (n = 10) *Kkay* + MI (n = 22)	~25 ~23 ~31.5 ~25 (from graph)	50 ± 4% 49 ± 2%	↓↓ ↓ (7 days PI)	70% 45%	Macrophages ↑↑, CD40 ↑↑,eotaxin ↑, EGF ↓↓, MDC ↑↑ myoglobin ↓↓, SGOT ↓↓, Oncostatin M, VWF ↓↓ Macrophages ↑, CD40 =, eotaxin ↑↑, EGF ↓↓↓, MDC =, myoglobin ↓, SGOT =, Oncostatin M =, VWF ↓
Feng et al., 2022 [59]	*db*/*db* *Kkay*	Mitsugumin 53 (**MG53**) protects diabetic hearts from I/R injury and ameliorates diet-induced cardiometabolic damage	male & female	n = 90	10 weeks	I/R 30 min I	db/+ + hSa (n = 8) db/+ + rhMG53-WT (n = 10) db/+ + rhMG53-C14A (n = 10) *db*/*db* + hSa (n = 10) *db*/*db* + rhMG53-WT (n = 12) *db*/*db* + rhMG53-C14A (n = 12) *Kkay* + hSa (n = 9) *Kkay* + rhMG53-WT (n = 10) *Kkay* + rhMG53-C14A (n = 9)	/	36.7% <14% <14% ~47% ~35% ~23% / / /	↓↓ ↓ ↓ ↓↓ ↓↓↓ ↓ (24 h PI)	/ / / 79% 48% 80%4 / / /	\
Honda et al., 2008 [70]	*Kkay*	Metabolic disorders exacerbate I/R injury as a result of **overexpression of inflammatory mediators**, and this effect might be improved by the anti-inflammatory effects of the **thiazolidinedione, pioglitazone**	male	n = 44	8–10 weeks	I/R 40 min I	WT (n = 10) WT + vehicle (n = 6) WT + Pioglitazone (n = 6) *Kkay* (n = 10) *Kkay* + vehicle (n = 6) *Kkay* + Pioglitazone (n = 6)	38.1 ± 1.3 21.4 ± 0.5 34.3 ± 0.8 20.2 ± 0.3 34.8 ± 0.7 20.6 ± 0.3	19.6 ± 2.5% 17.9 ± 1.4% 16.7 ± 2.6% 45.3 ± 2.7% 36.3 ± 3.4% 14.8 ± 4.6% (%AAR, 3 days PI)	/	/	Gr-1-+ granulocytes ↑, FA-11+macrophages ↑↑, MCP-1, KC, MIP-2, TNF-α, IL-10, MMP-9, TIMP-1, thioredoxin-1 ↑ / / Gr-1-+ granulocytes ↑↑↑, FA-11+macrophages ↑↑↑ MCP-1, KC, MIP-2, TNF-α, IL-10, MMP-9, TIMP-1, thioredoxin-1 ↑↑↑ Gr-1-+ granulocytes ↑↑, FA-11+macrophages ↑↑↑ MCP-1, KC, MIP-2, TNF-α, IL-10, MMP-9, TIMP-1, thioredoxin-1 ↑↑ Gr-1-+ granulocytes ↑, FA-11+macrophages ↑↑ MCP-1, KC, MIP-2, TNF-α, IL-10, MMP-9, TIMP-1, thioredoxin-1 ↑

* The tissue utilized in these studies arose from the same mice.

### 3.1. Ob/Ob Mice

The *ob*/*ob* mice were reported to have a larger infarct size, increased hypertrophy, decreased LV contractility, and worse outcome compared to wild-type (WT) mice after MI [53,58,62,64,65,66]. Importantly, obesity interferes with cardioprotective mechanisms such as pre- and postconditioning following MI [64]. Bouhidel et al. reported that ischemic postconditioning (IpostC) in *ob*/*ob* mice failed to reduce infarct size in contrast to the observed cardioprotective effect in healthy animals [48]. Attenuation of the reperfusion injury salvage kinase (RISK) pathway, which plays an important role in IpostC, might partly explain this loss of cardioprotection as *ob*/*ob* mice fail to activate and phosphorylate prosurvival kinases Akt, ERK1/2, pS6K1, and AMPK [53]. In contrast to these findings, Oosterlinck et al. reported that IpostC reduces infarct size and improves cardiac function in *ob*/*ob* mice and that this sustained cardioprotective effect resulted in increased survival. The divergent outcome might relate to differences in IPostC protocol, anesthesia, or gender differences in the studied population [58]. Another study showed that regular treadmill exercise in *ob*/*ob* mice significantly reduces infarct size by restoring the prosurvival signaling pathways with increased kinase phosphorylation. Regular exercise induced a strong cardioprotective effect in both WT and *ob*/*ob* mice, with no significant difference in infarct size between the two models. However, exercise did not alter hyperglycemia, hypercholesterolemia, hyperinsulinemia, adiposity, or bodyweight in *ob*/*ob* mice [64]. Cardioprotective effects are not always maintained in *ob*/*ob* mice, as demonstrated in a study involving N,N,N-Trimethylsphingosine chloride (TMS) treatment, a potent anti-inflammatory agent that blocks leukocyte activation and trans-endothelial migration of neutrophils. In this study, the cytoprotective effect of TMS after MI observed in WT mice was reduced in *ob*/*ob* mice [62]. Leptin signaling, among other factors, has been proven to play a crucial cardioprotective role after MI or I/R. Enhanced leptin signaling decreases cardiac apoptosis and reduces cardiac morbidity and mortality [65,66].

### 3.2. DIO Mice

Similar to their genetic variant, diet-induced obese (DIO) mice generally exhibit a larger infarct size and reduced survival compared to WT mice [55,67,68]. Thakker et al. demonstrated that DIO mice feature hyperinsulinemia, insulin resistance, and an altered inflammatory response after MI characterized by higher levels of pro-inflammatory chemokines and cytokines, increased macrophage density, and reduced neutrophil density compared to WT mice. This enhanced inflammatory profile was associated with adverse LV remodeling [55]. Some preclinical MI data in DIO mice also support the so-called obesity paradox [67,69,71]. Inserte et al. reported that the reduced infarct size in DIO mice correlated with a delay in intracellular pH normalization after reperfusion. However, this cardioprotective effect varied depending on the mouse strain [71]. Another study also observed improved myocardial tolerance to I/R injury, with better preserved left ventricular (LV) function. This improvement was linked to enhanced activation of the RISK pathway with increased phosphorylation of Akt and GSK3β [69]. Mouton et al. reported reduced survival following MI in DIO mice compared to chow-fed controls, with survival further reduced by angiotensin-induced hypertension. However, they observed improved post-MI cardiac function and remodeling in surviving normotensive DIO mice compared to the chow-fed group. Hypertension reversed this beneficial effect, leading to detrimental impacts on cardiac function and remodeling post-MI [67].

### 3.3. Db/Db Mice

Diabetes, another comorbidity associated with increased risk of MI, leads to poorer outcomes following MI in patients [72,73]. However, preclinical results in diabetic rodent models are rather inconsistent, reporting both improved and worse outcomes. Nevertheless, the majority of studies indicate that diabetes negatively interferes with cardioprotective mechanisms, which influences the outcome of therapeutic interventions [73]. The *db*/*db* mouse, an animal model for type 2 diabetes mellitus, exhibited a larger infarct size and reduced survival following myocardial I/R [56,59,61,63]. Jones et al. reported that the exacerbated I/R injury appears to be partly the result of an enhanced inflammatory response mediated by neutrophils with significantly increased accumulation of polymorphonuclear neutrophils (PMNs) in the diabetic heart after myocardial I/R compared to WT hearts [56]. In another study, *db*/*db* mice were subjected to 30, 45, and 60 min of ischemia, which showed a significant decrease in survival with increasing ischemia duration compared to non-diabetic mice [63]. Additionally, *db*/*db* mice subjected to 45 min of ischemia showed significant LV dilation, cardiac hypertrophy, and cardiac contractile dysfunction [63]. Similar to obese mice, the cardioprotective effects of TMS treatment are lost in *db*/*db* mice compared to WT mice [62]. Mechanisms contributing to myocardial dysfunction in *db*/*db* mice include increased fatty acid oxidation, increased ROS levels, mitochondrial dysfunction, enhanced inflammation, and impaired Ca^2+^ handling [74]. Further studies by Chou et al. demonstrated an increase in ventricular tachyarrhythmias following MI in *db*/*db* mice compared to controls. This increased arrhythmogenicity could be mitigated by reducing intracellular Ca^2+^ overload and oxidative stress [61]. Additional research showed that impaired cardiomyocyte calcium cycling in *db*/*db* mice is compensated by increased myofilament calcium sensitivity and increased titin-based stiffness prior to MI. In contrast, the sarcomere function of the remote myocardium is impaired after I/R due to the failure of both compensatory mechanisms and further depressed myocyte calcium handling [60].

### 3.4. KKAy and DKO Mice

Another murine model, the KKAy mouse, heterozygous for the yellow spontaneous mutation (*A^y^*) on a KK strain background, develops polygenic diabetes and shows hyperglycemia, hyperinsulinemia, glucose intolerance, and obesity [75]. This model serves as a valuable representation of metabolic syndrome. Honda et al. reported a significantly larger infarct size and increased inflammation in the ischemic myocardium in KKAy mice compared to WT. The KKay mice exhibited higher expression of chemokines, inflammatory cytokines, and extracellular matrix proteins in the ischemic region. These inflammatory mediators may exacerbate I/R injury, and this effect might be improved by anti-inflammatory interventions such as pioglitazone [70]. In contrast, Heaberlin et al. showed improved cardiac function but increased mortality in KKAy mice despite similar infarct size. This enhanced functionality was associated with reduced inflammation, including lower levels of macrophages, decreased extracellular matrix accumulation, and reduced neovascularization in the infarcted region of the KKAy mice [57]. An important factor contributing to the observed differences in the KKAy model is the variation in experimental protocols. Honda et al. utilized an I/R model (40 min of ischemia) [70], whereas Heaberlin et al. employed a permanent ligation model [57]. As previously described, reperfusion attenuates the inflammatory response [3,6,8,10], which may explain the increased infiltration of inflammatory cells and extracellular matrix proteins in the I/R model. This is likely the reason for the differential outcomes observed in these studies. The DKO mouse model reflects multiple significant comorbidities, including obesity, diabetes, dyslipidemia, and atherosclerosis. However, little data is reported on CVD and MI in this model. Similar to the observation in *ob*/*ob* mice by Oosterlinck et al. [58], DKO mice with metabolic syndrome had a larger infarct size than WT mice, but the cardioprotective effect of IPostC was maintained. Following IPostC, DKO mice showed a reduced infarct size and improved cardiac function, contributing to a decreased mortality [58].

## 4. Inflammatory Profiles of Obese and Diabetic Mouse Models

The analysis of the inflammatory processes in the mouse models is generally limited to the cytoprotective pathways and general analysis of leukocyte activation (Table 1). To gain a deeper understanding of the underlying mechanisms of the inflammatory response following MI, it is essential to investigate the interactions between these pathways. Therefore, it is crucial to have a comprehensive understanding of the inflammatory profiles of the patient population at risk and the relevant associated animal models.

Obesity is an important comorbidity in patients with ischemic heart disease and is associated with chronic systemic inflammation that can lead to type 2 diabetes with insulin resistance and β-cell dysfunction. This enhanced inflammation interferes with the inflammatory processes following MI and increases mortality and the development of heart failure after MI (Figure 2) [14]. Inflammation in obesity is characterized by increased NF-κB nuclear binding and elevated levels of adiponectin, cytokines (TNF-α, IL-1β, or IL-6), and chemokines, secreted from adipocytes and macrophages. Infiltrating macrophages exacerbate inflammation in white adipose tissue [14,76]. Since inflammation is a key player in cardioprotection following MI and I/R, a better understanding of the inflammasome and immune response in animal models is of paramount importance.

As previously described, the two most commonly used animal models to study obesity and related disorders are the *ob*/*ob* and *db*/*db* mice [55]. The disrupted leptin signaling in *ob*/*ob* and *db*/*db* mice, resulting from leptin deficiency and leptin receptor deficiency, respectively, impacts the immune response in these mouse models [77,78,79]. Leptin-deficient *ob*/*ob* and DIO mice show increased oxidative stress and enhanced systemic inflammation. In both these obese mouse models, as well as in obese patients, levels of MCP-1, also known as CCL2, are elevated. In mice, MCP-1 has been demonstrated to stimulate macrophage recruitment and to promote inflammation, glucose intolerance, and insulin insensitivity [77,80]. Because inherent immune-modulating abnormalities in genetic *ob*/*ob* and *db*/*db* mice need to be considered when studying inflammation related to cardiac repair after MI, Thakker et al. [55] have proposed a diet-induced mouse model of obesity (DIO) with an intact immune system as an alternative [55]. It has been demonstrated that DIO mice exhibited elevated levels of leptin, IL-6, KLF4, and STAT3. Additionally, DIO mice presented with increased levels of inflammatory cytokines and key immune cells, including Th17, M1 macrophages, and CD103+ dendritic cells, and reduced levels of PPAR-γ and Tregs, which might sustain adipose tissue inflammation [81,82]. In leptin-receptor-deficient *db*/*db* mice, both the innate and adaptive immune systems have been found to be altered. Specifically, *db*/*db* mice exhibited a significant increase in innate immune-inflammatory parameters, including macrophages, pro-inflammatory cells, and natural killer (NK) cells. Neutrophils also appear to play a critical role, as reported by Jones et al., following ischemia/reperfusion (I/R) in *db*/*db* mice [56]. The important role of neutrophils in MI was further demonstrated by Veltman et al. [7] in non-diabetic mice, who showed that inhibition of CLEC4E signaling, also known as Mincle, significantly reduced neutrophil infiltration, which was associated with decreased myocardial injury and improved LV structural and functional remodeling [7].

In contrast, the adaptive immune system, evaluated by mitogen-induced T and B cell proliferation and cytokine release, was significantly suppressed. In the spleen of *db*/*db* mice, fewer effector T cells but more regulatory T cells were observed [76]. Additionally, gene expression of inflammatory cytokines, including IL-1β, in peripheral leukocytes has been linked to the progression of diabetes over time in *db*/*db* mice [83]. Some studies also demonstrated that the NLRP3 inflammasome plays a role in adipose tissue and the liver, with its activation and the secretion of IL-1β, primarily attributed to infiltrating macrophages in these tissues [84].

Suriano et al. conducted an extensive comparison between the *ob*/*ob* and *db*/*db* mice in which they found distinct inflammatory profiles between both mouse models [85,86]. *ob*/*ob* mice presented with a higher inflammatory response in the liver, in comparison to a higher inflammatory tone in the (subcutaneous) adipose tissue for *db*/*db* mice. Expression of CCL2 (regulating migration and infiltration of monocytes), Adgre1 (mature macrophages), Itgax (dendritic cells), and CD68 (monocytes /macrophages) was significantly upregulated in the liver of *ob*/*ob* mice compared to *db*/*db* mice. This enhanced immune response was in line with the increased expression of related receptors, including TLR4, TLR2, and pro-inflammatory cytokines (TNFα, IL-1β). In contrast, *db*/*db* mice exhibited a significantly higher expression of CCL2, Adgre1, and CD68 in the subcutaneous adipose tissue. The increased macrophage infiltration was further confirmed by an increased number of crown-like structures (CLS), which have been associated with inflammation in obese humans and murine models. In the visceral adipose tissue, only IL-6 was significantly upregulated in *db*/*db*. Additionally, TNFα and IL-1β, but also Ptgs2 (coding for prostaglandin-endoperoxidase synthase 2), were also significantly increased in adipose tissue of *db*/*db* mice compared to *ob*/*ob* mice [86]. Additionally, *db*/*db* mice present a higher inflammatory state in the intestine as compared to *ob*/*ob* mice [85]. Although mice are generally classified as high-density lipoprotein models, significant strain-specific differences exist in lipoprotein metabolism and susceptibility to inflammatory stimuli. For example, C57Bl/6 mice display a polarization towards a Th1 profile with T cells mainly producing Th1 cytokines, particularly IFN-γ, while other strains, like BALB/c, produce more Th2 cytokines and less IFN-γ [87]. Considering the varying inflammatory profiles and immune responses of these mouse models following MI, careful consideration must be taken when selecting the appropriate animal model. Additionally, emerging evidence indicates sex-related differences in cardiac and inflammatory responses in murine models, highlighting the necessity of accounting for sex-related differences in experimental design and interpretation [35,67,73]. Female mice have been reported to exhibit increased resistance to myocardial ischemia-induced injury, resulting in lower mortality, reduced infarct size, and better-preserved cardiac function following MI compared to male mice [32,45,88]. Differences between females and males arise from multiple factors, including variations in leukocyte function, adiposity, immune metabolism, gene expression, and sex hormone levels. In murine models of MI, male and female mice show distinct inflammatory responses. Female mice exhibit higher reparative monocyte and macrophage populations, increased dendritic cell infiltration at both acute and chronic phases, and elevated inflammatory cytokines within the infarcted heart, supporting faster inflammation resolution and improved cardiac repair [89]. However, most studies reporting these inflammatory differences were conducted in young, healthy mice. It therefore remains unclear whether these sex-related inflammatory responses extend to aged mice and to models with comorbidities such as obesity. Taken together, all these differences in cardiac and inflammatory profiles across sexes and models should be accounted for when interpreting results to enhance translational outcomes.

## 5. Targeting Inflammation Following Myocardial Infarction

Myocardial healing, especially the acute inflammatory response, is a key determinant for the final infarct size and the subsequent risk of mortality and heart failure, marking inflammation as a strategic target for therapy [90]. As previously described, the innate immune response to reperfusion injury involves multiple immune cell types with ambivalent actions in different phases of the myocardial healing process. This complexity challenges our understanding and complicates the use of both narrow and broad-spectrum anti-inflammatory therapies [6,91].

Glucocorticoids and nonsteroidal anti-inflammatory drugs exemplify broad-acting anti-inflammatory agents that showed promise in preclinical models but failed to demonstrate clinical efficacy [6,13]. Likewise, cyclosporine A, an immunosuppressant, yielded inconsistent results in both animal and patient studies. These disappointing outcomes reflect the ambivalent roles of immune cells, where indiscriminate suppression can disrupt both damaging and reparative processes [6].

In contrast, more targeted strategies aim to modulate specific components of the immune response. Complement inhibition, chemokine blockade, and anti-adhesion therapies demonstrated cardioprotection in animal models, yet clinical trials yielded neutral or mixed results [6,90,91]. More encouraging data come from interventions targeting cytokine signaling. IL-1β inhibition, through agents like anakinra and canakinumab, has shown reductions in circulating inflammatory biomarker levels and adverse events. The CANTOS trial demonstrated that canakinumab lowered cardiovascular event rates in post-MI patients, while anakinra showed benefit in smaller studies such as VCU-ART3 [13]. Colchicine, evaluated in the COLCOT and LoDoCo2 trials, reduced major adverse cardiovascular events and inflammatory markers, though infarct size remained unaffected. IL-6 blockade with tocilizumab reduced myocardial injury in patients with STEMI [6,13,92].

Clinical translation remains a persistent challenge. This stems from the complexity of the immune response, which is temporally dynamic and pleiotropic, making selective targeting difficult. Preclinical animal models further complicate this picture as they rarely reflect the typical patient due to the lack of comorbidities and concomitant comedications. Finally, most therapeutic strategies discussed above focus on single immune components, overlooking the interconnected nature of the inflammatory cascade [6,90].

## 6. Imaging Inflammation

Inflammation is typically assessed using ex vivo methods such as histology, immunohistochemistry, and molecular assays, which provide detailed insights into cellular and molecular mechanisms. However, these techniques are inherently limited to single time points and require large numbers of animals to capture dynamic processes. In contrast, non-invasive imaging approaches allow for longitudinal serial assessments of both cardiac function and inflammatory processes within the same subjects, enhancing statistical power while significantly reducing the number of animals needed. This advantage is particularly critical for studying chronic inflammatory conditions, such as those associated with obesity and related metabolic disorders, where inflammation plays a central role in disease progression and outcomes following therapeutic interventions [93,94,95,96,97,98,99].

To gain a better understanding of these pro-inflammatory conditions in cardiac repair after AMI, in vivo imaging has become an indispensable tool. Non-invasive molecular and cellular imaging techniques enable researchers to monitor inflammatory processes over time and evaluate the efficacy of treatments in a physiologically relevant context. Among the available imaging modalities, positron emission tomography (PET) and magnetic resonance imaging (MRI) have emerged as the most prominent for preclinical cardiac molecular imaging.

PET relies on the use of radiolabeled tracers and is a highly sensitive imaging modality used to detect and quantify inflammation in various tissues, including the myocardium. This allows for localization and quantification of inflammation. One of the most used tracers is fluorodeoxyglucose (^18^F-FDG). FDG-PET capitalizes on the increased glucose uptake by activated inflammatory cells, particularly leukocytes, highlighting regions of active inflammation. However, highly metabolic cardiomyocytes can also generate a high FDG signal, potentially masking and/or misleading inflammatory activity. To overcome this limitation, novel radiotracers have been developed. Among others, they include ^68^Ga-pentixafor, which targets the C-X-X chemokine receptor 4 (CXCR4), and ^68^Ga-DOTATATE and ^68^Ga-DOTATOC, which characterize somatostatin receptor types 2 and 5 (SSTR2/5), respectively, focusing on pro-inflammatory macrophages. Additionally, ^11^C-methionine is another alternative used for imaging inflammation by targeting L-type amino acid transporter (LAT) as amino acids are largely metabolized by activated leukocytes [100,101,102,103].

In addition to PET, ionizing radiation-free MRI is commonly used due to its high resolution, enabling detailed visualization of cardiac tissue edema and fibrosis, which are indicative of inflammation. Traditional proton MRI (^1^H MRI) provides high anatomical detail and is the gold standard for assessing cardiac function [104]. ^1^H MRI also extends the possibility of tissue characterization via T_1_/T_2_ relaxation mapping, while newer techniques, including ^19^F-MRI, offer specific advantages in tracking inflammatory cells. ^19^F-MRI in combination with anatomical ^1^H-MRI is a powerful technique for imaging inflammation due to the absence of background signals from endogenous fluorine in biological tissues [105]. For example, fluorine-19 labeled compounds can be injected into the bloodstream, taken up by circulating macrophages, and accumulate in inflamed myocardial regions. The presence of these ^19^F-labeled tracers in inflamed tissues generates distinct signals detectable by MRI, enabling the visualization and quantification of inflammatory processes. Another recent study reported the generation of fluorine-loaded nanotracers specifically targeting either murine or human neutrophils, allowing non-invasive mapping and visualization of the systemic neutrophil dynamics upon cardiovascular injury [106].

Nanoparticles, such as ultrasmall superparamagnetic iron oxide (USPIO) and micron-sized particles of iron oxide (MPIO), are increasingly used in MRI to image inflammation due to their unique magnetic properties. Similar to fluorinated nanoparticles, USPIO and MPIO accumulate in areas of inflammation due to their uptake by macrophages (passive targeting) or targeted binding (active targeting). Accumulation of USPIOs and MPIOs alters the local magnetic environment, causing a decrease in signal intensity on T_2_/T_2_*-weighted images, highlighting inflamed regions with greater clarity. By targeting specific biomarkers of inflammation, such as vascular cell adhesion molecule-1 (VCAM-1), these nanoparticles can be functionalized to improve the specificity of molecular imaging. This targeted approach allows for the precise localization and quantification of inflammation, facilitating early diagnosis, monitoring of disease progression, and evaluation of therapeutic responses [107,108,109,110].

Cardiac PET-MRI combines the metabolic sensitivity of PET with the high-resolution anatomical and functional imaging capabilities of MRI. This hybrid approach simultaneously provides a comprehensive evaluation of cardiac function, myocardial inflammation, and related pathological processes. A key benefit is the ability to gain detailed insights into cardiovascular disease mechanisms and treatment responses in a single session, with efficient integration of data from both modalities [111,112].

Despite the many advantages of preclinical imaging modalities in visualizing inflammatory processes in vivo, certain challenges should be considered regarding data interpretation and reproducibility. Reproducibility remains a key challenge in preclinical imaging, largely due to the lack of standardized protocols for data acquisition and analysis needed to ensure interlaboratory consistency. In molecular imaging, quantitative accuracy is further affected by biological variability, different tracer kinetics, and technical variability across imaging platforms, all of which are amplified by the absence of universally accepted methodological standards. Limitations in achieving both high sensitivity and spatial resolution with single-modality imaging have led to the emergence of combined systems such as PET/MRI [113,114]. Despite these challenges, continued advances in imaging technology, tracer development, and data standardization are steadily improving the accuracy and translational relevance of preclinical inflammation imaging [98,99,101].

## 7. Conclusions and Future Perspectives

To improve clinical translation of preclinical findings, careful selection of representative animal models for studying CVD is crucial. Obese patients represent a significant portion of those affected by MI, and both obese patients and mouse models exhibit chronic systemic inflammation, which markedly influences the outcome of interventions. However, current data on MI and I/R in mouse models of obesity and related disorders are limited and often controversial. At the level of underlying mechanisms, the focus has been primarily on cytoprotective pathways. However, it is imperative to further investigate the interconnections between these pathways and the potential for pharmaceutical interventions. Understanding the distinct inflammatory signaling pathways in these models is vital for consistency in preclinical results and better clinical translation.

Future research must (I) address contradictory results related to the obesity paradox and postconditioning, (II) develop standardized protocols for MI (I/R) models, including for imaging data acquisition and processing, and (III) focus on mouse models that better represent at-risk patient populations. Investigating the early inflammatory phase, using innovative in vivo imaging modalities, in these models will undoubtedly increase our understanding of the inflammatory processes involved. Additionally, considering comorbidities such as pro-inflammatory epicardial adipose tissue, investigating gender differences, and exploring therapeutics targeting specific inflammatory pathways are all essential. Addressing these knowledge gaps will enhance our understanding of the interactions between obesity, inflammation, and MI, leading to more effective interventions and better patient outcomes.

## Data Availability

No new data were created or analyzed in this study. Data sharing is not applicable to this article.

## Abbreviations

CVD	Cardiovascular Disease
DAMPs	Damage-Associated Molecular Patterns
DIO	Diet-Induced Obese
IpostC	Ischemic Postconditioning
I/R	Ischemia–Reperfusion
LAD	Left Anterior Descending Artery
LV	Left Ventricle
MI	Myocardial Infarction
PCI	Percutaneous Coronary Intervention
RISK	Reperfusion Injury Salvage Kinase
WT	Wild-Type

## References

[1] Salari N., Morddarvanjoghi F., Abdolmaleki A., Rasoulpoor S., Khaleghi A.A., Hezarkhani L.A., Shohaimi S., Mohammadi M. (2023). The global prevalence of myocardial infarction: A systematic review and meta-analysis. BMC Cardiovasc. Disord..

[2] Laforgia P.L., Auguadro C., Bronzato S., Durante A. (2022). The Reduction of Mortality in Acute Myocardial Infarction: From Bed Rest to Future Directions. Int. J. Prev. Med..

[3] Algoet M., Janssens S., Himmelreich U., Gsell W., Pusovnik M., Eynde J.V.D., Oosterlinck W. (2023). Myocardial ischemia–reperfusion injury and the influence of inflammation. Trends Cardiovasc. Med..

[4] Elgendy I.Y., Mahtta D., Pepine C.J. (2019). Medical Therapy for Heart Failure Caused by Ischemic Heart Disease. Circ. Res..

[5] Ibanez B., James S., Agewall S., Antunes M.J., Bucciarelli-Ducci C., Bueno H., Caforio A.L.P., Crea F., Goudevenos J.A., Halvorsen S. (2018). 2017 ESC Guidelines for the management of acute myocardial infarction in patients presenting with ST-segment elevation. Eur. Heart J..

[6] Francisco J., Del Re D.P. (2023). Inflammation in Myocardial Ischemia/Reperfusion Injury: Underlying Mechanisms and Therapeutic Potential. Antioxidants.

[7] Veltman D., Wu M., Pokreisz P., Claus P., Gillijns H., Caluwé E., Vanhaverbeke M., Gsell W., Himmelreich U., Sinnaeve P.R. (2021). Clec4e-Receptor Signaling in Myocardial Repair After Ischemia–Reperfusion Injury. JACC Basic Transl. Sci..

[8] Zhang R.Y.K., Cochran B.J., Thomas S.R., Rye K. (2023). Impact of Reperfusion on Temporal Immune Cell Dynamics After Myocardial Infarction. J. Am. Heart Assoc..

[9] Frangogiannis N.G., Smith C.W., Entman M.L. (2002). The inflammatory response in myocardial infarction. Cardiovasc. Res..

[10] Adrie C., Shin S.A., Monchi M., Cavaillon J.M. (2017). Ischemia–Reperfusion Syndrome. Inflammation: From Molecular and Cellular Mechanisms to the Clinic.

[11] Zhang H., Hu H., Zhai C., Jing L., Tian H. (2023). Cardioprotective Strategies After Ischemia–Reperfusion Injury. Am. J. Cardiovasc. Drugs.

[12] Li T., Yan Z., Fan Y., Fan X., Li A., Qi Z., Zhang J. (2023). Cardiac repair after myocardial infarction: A two-sided role of inflammation-mediated. Front. Cardiovasc. Med..

[13] Matter M.A., Paneni F., Libby P., Frantz S., Stähli B.E., Templin C., Mengozzi A., Wang Y.-J., Kündig T.M., Räber L. (2023). Inflammation in acute myocardial infarction: The good, the bad and the ugly. Eur. Heart J..

[14] Rohm T.V., Meier D.T., Olefsky J.M., Donath M.Y. (2022). Inflammation in obesity, diabetes, and related disorders. Immunity.

[15] Powell-Wiley T.M., Poirier P., Burke L.E., Després J.-P., Gordon-Larsen P., Lavie C.J., Lear S.A., Ndumele C.E., Neeland I.J., Sanders P. (2021). Obesity and Cardiovascular Disease: A Scientific Statement from the American Heart Association. Circulation.

[16] Thomsen M., Nordestgaard B.G. (2014). Myocardial Infarction and Ischemic Heart Disease in Overweight and Obesity with and Without Metabolic Syndrome. JAMA Intern. Med..

[17] Lassale C., Tzoulaki I., Moons K.G.M., Sweeting M., Boer J., Johnson L., Huerta J.M., Agnoli C., Freisling H., Weiderpass E. (2018). Separate and combined associations of obesity and metabolic health with coronary heart disease: A pan-European case-cohort analysis. Eur. Heart J..

[18] Piché M.-E., Tchernof A., Després J.-P. (2020). Obesity Phenotypes, Diabetes, and Cardiovascular Diseases. Circ. Res..

[19] Lempesis I.G., Georgakopoulou V.E. (2023). Physiopathological mechanisms related to inflammation in obesity and type 2 diabetes mellitus. World J. Exp. Med..

[20] Battineni G., Sagaro G.G., Chintalapudi N., Amenta F., Tomassoni D., Tayebati S.K. (2021). Impact of Obesity-Induced Inflammation on Cardiovascular Diseases (CVD). Int. J. Mol. Sci..

[21] Antonopoulos A.S., Papastamos C., Cokkinos D.V., Tsioufis K., Tousoulis D. (2023). Epicardial Adipose Tissue in Myocardial Disease: From Physiology to Heart Failure Phenotypes. Curr. Probl. Cardiol..

[22] Kruszewska J., Cudnoch-Jedrzejewska A., Czarzasta K. (2022). Remodeling and Fibrosis of the Cardiac Muscle in the Course of Obesity—Pathogenesis and Involvement of the Extracellular Matrix. Int. J. Mol. Sci..

[23] Feng Q., Li Q., Zhou H., Sun L., Lin C., Jin Y., Wang D., Guo G. (2023). The role of major immune cells in myocardial infarction. Front. Immunol..

[24] Lavie C.J., McAuley P.A., Church T.S., Milani R.V., Blair S.N. (2014). Obesity and cardiovascular diseases: Implications regarding fitness, fatness, and severity in the obesity paradox. J. Am. Coll. Cardiol..

[25] Kanic V., Vollrath M., Frank B., Kanic Z. (2021). An obesity paradox in patients with myocardial infarction undergoing percutaneous intervention. Nutr. Metab. Cardiovasc. Dis..

[26] Ndrepepa G., Kastrati A. (2020). Obesity paradox after percutaneous coronary intervention-Closing in on the truth behind the phe-nomenon. EuroIntervention.

[27] Aggrawal K., Gupta V., Singh B., Medatwal R., Singh S., Jain P., Jain R. (2025). Exploring the obesity parADOX: A multisystem review. Am. J. Med. Sci..

[28] Butt J.H., Petrie M.C., Jhund P.S., Sattar N., Desai A.S., Køber L., Rouleau J.L., Swedberg K., Zile M.R., Solomon S.D. (2023). Anthropometric measures and adverse outcomes in heart failure with reduced ejection fraction: Revisiting the obesity paradox. Eur. Heart J..

[29] Chang V.W., Langa K.M., Weir D., Iwashyna T.J. (2017). The obesity paradox and incident cardiovascular disease: A population-based study. PLoS ONE.

[30] De Schutter A., Kachur S., Lavie C.J., Boddepalli R.S., Patel D.A., Milani R.V. (2016). The impact of inflammation on the obesity paradox in coronary heart disease. Int. J. Obes..

[31] Jia T., Wang C., Han Z., Wang X., Ding M., Wang Q. (2020). Experimental Rodent Models of Cardiovascular Diseases. Front. Cardiovasc. Med..

[32] Martin T.P., Macdonald E.A., Ali A., Elbassioni M., O’toole D., Zaeri A.A.I., Nicklin S.A., Gray S.A., Loughrey G.M. (2022). Preclinical models of myocardial infarction: From mechanism to translation. Br. J. Pharmacol..

[33] Heusch G. (2020). Myocardial ischaemia–reperfusion injury and cardioprotection in perspective. Nat. Rev. Cardiol..

[34] Alfaddagh A., Martin S.S., Leucker T.M., Michos E.D., Blaha M.J., Lowenstein C.J., Jones S.R., Toth P.P. (2020). Inflammation and cardiovascular disease: From mechanisms to therapeutics. Am. J. Prev. Cardiol..

[35] De Villiers C., Riley P.R. (2020). Mouse models of myocardial infarction: Comparing permanent ligation and ischaemia-reperfusion. Dis. Model. Mech..

[36] Kane J.J., Murphy M.L., Bisset J.K., Desoyza N., Doherty J.E., Straub K.D. (1975). Mitochondrial function, oxygen extraction, epicardial S-T segment changes and tritiated digoxin distribution after reperfusion of ischemic myocardium. Am. J. Cardiol..

[37] Bresnahan G.F., Roberts R., Shell W.E., Ross J., Sobel B.E. (1974). Deleterious effects due to hemorrhage after myocardial reperfusion. Am. J. Cardiol..

[38] Kloner R.A., Ganote C.E., Jennings R.B. (1974). The “No-Reflow” Phenomenon after Temporary Coronary Occlusion in the Dog. J. Clin. Invest..

[39] Saÿen J.J., Sheldon W.F., Horwitz O., Kuo P.T., Peirce G., Zinsser H.F., Mead J. (1951). Studies of coronary disease in the experimental animal. Ii. Polarographic determinations of local oxygen availability in the dog’s left ventricle during coronary occlusion and pure oxygen breathing. J. Clin. Investig..

[40] Kreutzer F.P., Meinecke A., Schmidt K., Fiedler J., Thum T. (2022). Alternative strategies in cardiac preclinical research and new clinical trial formats. Cardiovasc. Res..

[41] Camacho P., Fan H., Liu Z., He J.-Q. (2016). Small mammalian animal models of heart disease. Am. J. Cardiovasc. Dis..

[42] Yeap X.Y., Dehn S., Adelman J., Lipsitz J., Thorp E.B. (2013). Quantitation of acute necrosis after experimental myocardial infarction. Methods Mol. Biol..

[43] Stone G.W., Selker H.P., Thiele H., Patel M.R., Udelson J.E., Ohman E.M., Maehara A., Eitel I., Granger C.B., Jenkins P.L. (2016). Relationship Between Infarct Size and Outcomes Following Primary PCI: Patient-Level ASnalysis From 10 Randomized Trials. J. Am. Coll. Cardiol..

[44] Heusch G., Gersh B.J. (2017). The pathophysiology of acute myocardial infarction and strategies of protection beyond reperfusion: A continual challenge. Eur. Heart J..

[45] Lindsey M.L., Brunt K.R., Kirk J.A., Kleinbongard P., Calvert J.W., Brás L.E., DeLeon-Pennell K.Y., Del Re D.P., Frangogiannis N.G., Frantz S. (2021). Guidelines for in vivo mouse models of myocardial infarction. Am. J. Physiol. Heart Circ. Physiol..

[46] Lindsey M.L., Bolli R., Canty J.M., Du X.-J., Frangogiannis N.G., Frantz S., Gourdie R.G., Holmes J.W., Jones S.P., Kloner R.A. (2018). Guidelines for experimental models of myocardial ischemia and infarction. Am. J. Physiol. Heart Circ. Physiol..

[47] Peet C., Ivetic A., Bromage D.I., Shah A.M. (2021). Cardiac monocytes and macrophages after myocardial infarction. Cardiovasc. Res..

[48] Heusch G. (2015). Molecular basis of cardioprotection signal transduction in ischemic pre-, post-, and remote conditioning. Circ. Res..

[49] Bromage D.I., Pickard J.M.J., Rossello X., Ziff O.J., Burke N., Yellon D.M., Davidson S.M. (2017). Remote ischaemic conditioning reduces infarct size in animal in vivo models of ischaemia-reperfusion injury: A systematic review and meta-analysis. Cardiovasc. Res..

[50] Kim S.C., Boehm O., Meyer R., Hoeft A., Knüfermann P., Baumgarten G.A. (2012). murine closed-chest model of myocardial ischemia and reperfusion. J. Vis. Exp..

[51] Algoet M., Pusovnik M., Gillijns H., Mestdagh S., Billiau J., Artoos I., Gsell W., Janssens S.P., Himmelreich U., Oosterlinck W. (2024). Remotely Triggered LAD Occlusion Using a Balloon Catheter in Spontaneously Breathing Mice. J. Vis. Exp..

[52] Sicklinger F., Zhang Y., Lavine K.J., Simon N., Bucher V., Jugold M., Lehmann L., Konstandin M.H., Katus H.A., Leuschner F. (2020). A Minimal-Invasive Approach for Standardized Induction of Myocardial Infarction in Mice. Circ. Res..

[53] Bouhidel O., Pons S., Souktani R., Zini R., Berdeaux A., Ghaleh B. (2008). Myocardial ischemic postconditioning against ischemia–reperfusion is impaired in *ob*/*ob* mice. Am. J. Physiol. Heart Circ. Physiol..

[54] Shin H.S., Shin H.H., Shudo Y. (2021). Current Status and Limitations of Myocardial Infarction Large Animal Models in Cardiovascular Translational Research. Front. Bioeng. Biotechnol..

[55] Thakker G.D., Frangogiannis N.G., Bujak M., Zymek P., Gaubatz J.W., Reddy A.K., Taffet G., Michael L.H., Entman M.L., Ballantyne C.M. (2006). Effects of diet-induced obesity on inflammation and remodeling after myocardial infarction. Am. J. Physiol. Heart Circ. Physiol..

[56] Jones S.P., Girod W.G., Granger D.N., Palazzo A.J., Lefer D.J. (1999). Reperfusion injury is not affected by blockade of P-selectin in the diabetic mouse heart. Am. J. Physiol. Circ. Physiol..

[57] Heaberlin J.R., Ma Y., Zhang J., Ahuja S.S., Lindsey M.L., Halade G.V. (2013). Obese and diabetic KKAy mice show increased mortality but improved cardiac function following myocardial infarction. Cardiovasc. Pathol..

[58] Oosterlinck W., Dresselaers T., Geldhof V., Nevelsteen I., Janssens S., Himmelreich U., Herijgers P. (2013). Diabetes mellitus and the metabolic syndrome do not abolish, but might reduce, the cardioprotective effect of ischemic postconditioning. J. Thorac. Cardiovasc. Surg..

[59] Feng H., Shen H., Robeson M., Wu Y., Wu H., Chen G., Zhang S., Xie P., Jin L., He Y. (2022). MG53 E3 Ligase–Dead Mutant Protects Diabetic Hearts from Acute Ischemic/Reperfusion Injury and Ameliorates Diet-Induced Cardiometabolic Damage. Diabetes.

[60] Funk F., Kronenbitter A., Isić M., Flocke V., Gorreßen S., Semmler D., Brinkmann M., Beck K., Steinhoff O., Srivastava T. (2022). Diabetes disturbs functional adaptation of the remote myocardium after ischemia/reperfusion. J. Mol. Cell. Cardiol..

[61] Chou C.C., Lee H.L., Chang G.J., Wo H.T., Yen T.H., Wen M.S., Chu Y., Liu H.T., Chang P.C. (2020). Mechanisms of ranolazine pretreatment in preventing ventricular tachyarrhythmias in diabetic *db*/*db* mice with acute regional ischemia–reperfusion injury. Sci. Rep..

[62] Gundewar S., Calvert J.W., Elrod J.W., Lefer D.J. (2007). Cytoprotective effects of N,N,N-trimethylsphingosine during ischemia–reperfusion injury are lost in the setting of obesity and diabetes. Am. J. Physiol. Circ. Physiol..

[63] Greer J.J.M., Ware D.P., Lefer D.J. (2006). Myocardial infarction and heart failure in the *db*/*db* diabetic mouse. Am. J. Physiol.-Heart Circ. Physiol..

[64] Pons S., Martin V., Portal L., Zini R., Morin D., Berdeaux A., Ghaleh B. (2013). Regular treadmill exercise restores cardioprotective signaling pathways in obese mice independently from improvement in associated co-morbidities. J. Mol. Cell. Cardiol..

[65] McGaffin K.R., Zou B., McTiernan C.F., O’dOnnell C.P. (2009). Leptin attenuates cardiac apoptosis after chronic ischaemic injury. Cardiovasc. Res..

[66] McGaffin K.R., Sun C.-K., Rager J.J., Romano L.C., Zou B., Mathier M.A., O’Doherty R.M., McTiernan C.F., O’Donnell C.P. (2008). Leptin signalling reduces the severity of cardiac dysfunction and remodelling after chronic ischaemic injury. Cardiovasc. Res..

[67] Mouton A.J., Flynn E.R., Moak S.P., Li X., da Silva A.A., Wang Z., Carmo J.M.D., Hall M.E., Hall J.E. (2021). Interaction of Obesity and Hypertension on Cardiac Metabolic Remodeling and Survival Following Myocardial Infarction. J. Am. Heart Assoc..

[68] Yoshii A., Nagoshi T., Kashiwagi Y., Kimura H., Tanaka Y., Oi Y., Ito K., Yoshino T., Tanaka T.D., Yoshimura M. (2019). Cardiac ischemia–reperfusion injury under insulin-resistant conditions: SGLT1 but not SGLT2 plays a compensatory protective role in diet-induced obesity. Cardiovasc. Diabetol..

[69] Poncelas M., Inserte J., Vilardosa Ú., Rodriguez-Sinovas A., Bañeras J., Simó R., Garcia-Dorado D. (2015). Obesity induced by high fat diet attenuates postinfarct myocardial remodeling and dysfunction in adult B6D2F1 mice. J. Mol. Cell. Cardiol..

[70] Honda T., Kaikita K., Tsujita K., Hayasaki T., Matsukawa M., Fuchigami S., Sugiyama S., Sakashita N., Ogawa H., Takeya M. (2008). Pioglitazone, a peroxisome proliferator-activated receptor-γ agonist, attenuates myocardial ischemia–reperfusion injury in mice with metabolic disorders. J. Mol. Cell. Cardiol..

[71] Inserte J., Aluja D., Barba I., Ruiz-Meana M., Miró E., Poncelas M., Vilardosa Ú., Castellano J., Garcia-Dorado D. (2019). High-fat diet improves tolerance to myocardial ischemia by delaying normalization of intracellular PH at reperfusion. J. Mol. Cell. Cardiol..

[72] Cui J., Liu Y., Li Y., Xu F., Liu Y. (2021). Type 2 Diabetes and Myocardial Infarction: Recent Clinical Evidence and Perspective. Front. Cardiovasc. Med..

[73] Ferdinandy P., Hausenloy D.J., Heusch G., Baxter G.F., Schulz R. (2014). Interaction of Risk Factors, Comorbidities, and Comedications with Ischemia/Reperfusion Injury and Cardioprotection by Preconditioning, Postconditioning, and Remote Conditioning. Pharmacol. Rev..

[74] Riehle C., Bauersachs J. (2019). Of mice and men: Models and mechanisms of diabetic cardiomyopathy. Basic Res. Cardiol..

[75] The Jackson Laboratory [Internet] (2024). KK.Cg-Ay/J..

[76] Lee S.E., Jang I.S., Park J.S., Lee J.H., Lee S.Y., Baek S.Y., Lee S.H., Lee H. (2010). Systemic immunity of obese-diabetes model (*db*/*db*) mice. Mol. Cell. Toxicol..

[77] Hornung F., Rogal J., Loskill P., Löffler B., Deinhardt-Emmer S. (2021). The Inflammatory Profile of Obesity and the Role on Pulmonary Bacterial and Viral Infections. Int. J. Mol. Sci..

[78] Adamowski M., Sharma Y., Molcan T., Wołodko K., Kelsey G., Galvão A.M. (2024). Leptin signalling regulates transcriptional differences in granulosa cells from genetically obese mice but not the activation of NLRP3 inflammasome. Sci. Rep..

[79] Wang B., Chandrasekera P.C., Pippin J.J. (2014). Leptin and Leptin Receptor-Deficient Rodent Models: Relevance for Human Type 2 Diabetes. Curr. Diabetes Rev..

[80] Frühbeck G., Catalán V., Rodríguez A., Ramírez B., Becerril S., Portincasa P., Gómez-Ambrosi J. (2017). Normalization of adiponectin concentrations by leptin replacement in *ob*/*ob* mice is accompanied by reductions in systemic oxidative stress and inflammation. Sci. Rep..

[81] Fenton J.I., Nuñez N.P., Yakar S., Perkins S.N., Hord N.G., Hursting S.D. (2009). Diet-induced adiposity alters the serum profile of inflammation in C57BL/6N mice as measured by antibody array. Diabetes Obes. Metab..

[82] Kiran S., Rakib A., Kodidela S., Kumar S., Singh U.P. (2022). High-Fat Diet-Induced Dysregulation of Immune Cells Correlates with Macrophage Phenotypes and Chronic Inflammation in Adipose Tissue. Cells.

[83] Fujimoto S., Mochizuki K., Goda T. (2010). Gene Expression of Inflammatory Cytokines in Peripheral Leukocytes in *db*/*db* Mice Rose with Progression of Diabetes. Biosci. Biotechnol. Biochem..

[84] Kammoun H., Allen T., Henstridge D., Barre S., Coll R., Lancaster G., Cron L., Reibe S., Chan J., Bensellam M. (2018). Evidence against a role for NLRP3-driven islet inflammation in *db*/*db* mice. Mol. Metab..

[85] Suriano F., Manca C., Flamand N., Depommier C., Van Hul M., Delzenne N.M., Silvestri C., Cani P.D., Di Marzo V. (2022). Exploring the endocannabinoidome in genetically obese (*ob*/*ob*) and diabetic (*db*/*db*) mice: Links with inflammation and gut microbiota. Biochim. Biophys. Acta (BBA)-Mol. Cell Biol. Lipids.

[86] Suriano F., Vieira-Silva S., Falony G., Roumain M., Paquot A., Pelicaen R., Régnier M., Delzenne N.M., Raes J., Muccioli G.G. (2021). Novel insights into the genetically obese (*ob*/*ob*) and diabetic (*db*/*db*) mice: Two sides of the same coin. Microbiome.

[87] Oppi S., Lüscher T.F., Stein S. (2019). Mouse Models for Atherosclerosis Research—Which Is My Line?. Front. Cardiovasc. Med..

[88] Shioura K.M., Geenen D.L., Goldspink P.H. (2008). Sex-related changes in cardiac function following myocardial infarction in mice. Am. J. Physiol.-Regul. Integr. Comp. Physiol..

[89] Pullen A.B., Kain V., Serhan C.N., Halade G.V. (2020). Molecular and Cellular Differences in Cardiac Repair of Male and Female Mice. J. Am. Heart Assoc..

[90] Andreadou I., Cabrera-Fuentes H.A., Devaux Y., Frangogiannis N.G., Frantz S., Guzik T., Liehn E.A., Gomes C.P.C., Schulz R., Hausenloy D.J. (2019). Immune cells as targets for cardioprotection: New players and novel therapeutic opportunities. Cardiovasc. Res..

[91] Frangogiannis N.G. (2014). The inflammatory response in myocardial injury, repair, and remodelling. Nat. Rev. Cardiol..

[92] Heusch G. (2024). Myocardial ischemia/reperfusion: Translational pathophysiology of ischemic heart disease. Med.

[93] Wachsmuth L., Mensen A., Barca C., Wiart M., Tristão-Pereira C., Busato A., Waiczies S., Himmelreich U., Millward J.M., Reimann H.M. (2021). Contribution of preclinical MRI to responsible animal research: Living up to the 3R principle. Magn. Reson. Mater. Phys. Biol. Med..

[94] Kiessling F., Pichler B.J., Hauff P. (2017). Small Animal Imaging: Basics and Practical Guide.

[95] Sosnovik D.E., Scherrer-Crosbie M. (2022). Biomedical Imaging in Experimental Models of Cardiovascular Disease. Circ. Res..

[96] Sinusas A.J., Bengel F., Nahrendorf M., Epstein F.H., Wu J.C., Villanueva F.S., Fayad Z.A., Gropler R.J. (2008). Multimodality Cardiovascular Molecular Imaging, Part, I. Circ. Cardiovasc. Imaging.

[97] Vandoorne K., Nahrendorf M. (2017). Multiparametric Imaging of Organ System Interfaces. Circ. Cardiovasc. Imaging.

[98] Macritchie N., Noonan J., Tomasz |., Guzik J., Maffia P. (2021). Molecular imaging of cardiovascular inflammation. Br. J. Pharmacol..

[99] MacRitchie N., Frleta-Gilchrist M., Sugiyama A., Lawton T., McInnes I.B., Maffia P. (2020). Molecular imaging of inflammation-Current and emerging technologies for diagnosis and treatment. Pharmacol. Ther..

[100] Heo G.S., Diekmann J., Thackeray J.T., Liu Y. (2023). Nuclear Methods for Immune Cell Imaging: Bridging Molecular Imaging and Individualized Medicine. Circ. Cardiovasc. Imaging.

[101] Thackeray J.T., Bengel F.M. (2018). Molecular Imaging of Myocardial Inflammation with Positron Emission Tomography Post-Ischemia: A Determinant of Subsequent Remodeling or Recovery. JACC Cardiovasc. Imaging.

[102] Thackeray J.T., Lavine K.J., Liu Y. (2023). Imaging Inflammation Past, Present, and Future: Focus on Cardioimmunology. J. Nucl. Med..

[103] Hess A., Derlin T., Koenig T., Diekmann J., Wittneben A., Wang Y., Wester H.J., Ross T.L., Wollert K.C., Bauersachs J. (2020). Molecular imaging-guided repair after acute myocardial infarction by targeting the chemokine receptor CXCR4. Eur. Heart J..

[104] Sosnovik D.E., Nahrendorf M., Weissleder R. (2007). Molecular Magnetic Resonance Imaging in Cardiovascular Medicine. Circulation.

[105] Flögel U., Ding Z., Hardung H., Jander S., Reichmann G., Jacoby C., Schubert R., Schrader J. (2008). In Vivo Monitoring of Inflammation After Cardiac and Cerebral Ischemia by Fluorine Magnetic Resonance Imaging. Circulation.

[106] Bouvain P., Ding Z., Kadir S., Kleimann P., Kluge N., Tiren Z.B., Steckel B., Flocke V., Zalfen R., Petzsch P. (2023). Non-invasive mapping of systemic neutrophil dynamics upon cardiovascular injury. Nat. Cardiovasc. Res..

[107] Strijkers G.J. (2013). Targeted Nanoparticles for Cardiovascular Molecular Imaging. Curr. Radiol. Rep..

[108] Stirrat C.G., Newby D.E., Robson J.M.J., Jansen M.A. (2014). The Use of Superparamagnetic Iron Oxide Nanoparticles to Assess Cardiac Inflammation. Curr. Cardiovasc. Imaging Rep..

[109] Tsampasian V., Merinopoulos I., Cameron D., Garg P., Vassiliou V.S. (2022). Ultrasmall Superparamagnetic Particles of Iron Oxide and Cardiac Magnetic Resonance: Novel Imaging in Everyday Conditions. Appl. Sci..

[110] Merinopoulos I., Gunawardena T., Stirrat C., Cameron D., Eccleshall S.C., Dweck M.R., Newby D.E., Vassiliou V.S. (2021). Diagnostic Applications of Ultrasmall Superparamagnetic Particles of Iron Oxide for Imaging Myocardial and Vascular Inflammation. JACC Cardiovasc. Imaging.

[111] Kazimierczyk R., Kaminski K.A., Nekolla S.G. (2024). Cardiac PET/MRI: Recent Developments and Future Aspects. Semin. Nucl. Med..

[112] Rischpler C., Siebermair J., Kessler L., Quick H.H., Umutlu L., Rassaf T., Antoch G., Herrmann K., Nensa F. (2020). Cardiac PET/MRI: Current Clinical Status and Future Perspectives. Semin. Nucl. Med..

[113] Strunk M., Heo G.S., Hess A., Luehmann H., Ross T.L., Gropler R.J., Bengel F.M., Liu Y., Thackeray J.T. (2024). Toward Quantitative Multisite Preclinical Imaging Studies in Acute Myocardial Infarction: Evaluation of the Immune-Fibrosis Axis. J. Nucl. Med..

[114] Gsell W., Molinos C., Correcher C., Belderbos S., Wouters J., Junge S., Heidenreich M., Velde G.V., Rezaei A., Nuyts J. (2020). Characterization of a preclinical PET insert in a 7 tesla MRI scanner: Beyond NEMA testing. Phys. Med. Biol..

